# High efficiency and large optical anisotropy in the high-order nonlinear processes of 2D perovskite nanosheets

**DOI:** 10.1515/nanoph-2021-0789

**Published:** 2022-03-01

**Authors:** Zehong Chen, Zhonghong Shi, Wenbo Zhang, Zixian Li, Zhang-Kai Zhou

**Affiliations:** State Key Laboratory of Optoelectronic Materials and Technologies, School of Physics, Sun Yat-sen University, Guangzhou 510275, China

**Keywords:** 2D perovskites, multiphoton photoluminescence, optical anisotropy, third-harmonic generation

## Abstract

Nonlinear nanophotonic devices have brought about great advances in the fields of nano-optics, quantum science, biomedical engineering, etc. However, in order to push these nanophotonic devices out of laboratory, it is still highly necessary to improve their efficiency. Since obtaining novel nanomaterials with large nonlinearity is of crucial importance for improving the efficiency of nonlinear nanodevices, we propose the two-dimensional (2D) perovskites. Different from most previous studies which focused on the 2D perovskites in large scale (such as the bulk materials or the thick flakes), herein we studied the 2D perovskites nanosheets with thickness of ∼50 nm. The high-order nonlinear processes including multi-photon photoluminescence and third-harmonic generation (THG) have been systematically investigated, and it is found the THG process can have a high conversion efficiency up to ∼8 × 10^−6^. Also, it is observed that the nonlinear responses of 2D perovskites have large optical anisotropy, i.e., the polarization ratio for the incident polarization dependence of nonlinear response can be as high as ∼0.99, which is an impressive record in the perovskite systems. Our findings reveal the properties of high efficiency and huge optical anisotropy in the nonlinear processes of 2D perovskite nanosheets, shedding light on the design of advanced integrated nonlinear nanodevices in future.

## 1 Introduction

Due to the remarkable performances in frequency conversion, entanglement generation, photon detection, as well as the small size easy for integration, nonlinear nanophotonic devices have brought about great advances to the fields of nano-optics, integrated optics, quantum science, biomedical engineering, etc. For example, the quantum source based on four waves mixing effects has greatly promoted the study of on-chip quantum photonic circuits [1, 2]; also, nanomaterials with high efficient multiphoton fluorescence have found important applications in bioimaging and biosensing [3].

Restricted by the requirement of subwavelength scale which offers the possibility for high integration, the size of material which generates the nonlinear process in nanophotonic device is usually very small, and therefore its optical nonlinear response is relatively weak (comparing with bulk nonlinear material). This fact leads to the low efficiency of nonlinear nanodevices, greatly limiting their future applications and developments. In order to address this problem, to obtain the materials which possess large nonlinearity even when they are in nanoscale is of crucial importance.

Two-dimensional (2D) organic–inorganic hybrid halide perovskite is a new type of semiconductor material with large exciton binding energy, high photoluminescence quantum efficiency, tunability of the photoluminescence wavelength, low-temperature solution processability [4], [5], [6], [7], as well as intense nonlinear responses including third-harmonic generation (THG) [8], multiphoton absorption [9], saturated absorption [10], etc. The 2D perovskites can mainly be divided into three types according to the longitudinal stacking of molecules layers: RP (Ruddlesden–Popper) phase [11], DJ (Dion–Jacobson) phase [12, 13] and ACI (alternating cation in the interlayer space) phase [14, 15]. Their general chemical formulas are A′_2_A_*n*−1_B_*n*_X_3*n*+1_, A′A_*n*−1_B_*n*_X_3*n*+1_, A′A_*n*_B_*n*_X_3*n*+1_, respectively, where A′ is a long-chain organic cation, A is a short-chain organic cation, and B is a metal cation (Pb, Sn), X is a halogen anion (Cl, Br, I). The organic spacer layer A′ existing in 2D perovskites forms a van der Waals structure similar to other 2D materials [16], which can be alternately arranged with the perovskite layer to form self-assemble multiple quantum wells (MQWs) [17]. The diversity of organic molecules and multiple substitutions of molecules in A′, A, B, X sites of the 2D perovskites chemical formulas make it possible to break the centrosymmetric restriction, leading to the second-harmonic generation (SHG) with high polarization ratio [18]. The various nonlinear effects supported by 2D perovskites provide abundant possibilities for future integrated photonic devices and other related applications, such as all-optical modulation [19], quasi-2D lasers [20, 21], photodetection [22], [23], [24], and terahertz photonics [25].

Despite of these achievements, it is found that the optical properties (including both linear and nonlinear) of 2D perovskite are mainly explored in the systems with large scale, such as the bulk materials or the flakes with thickness over 100 nm [11, 23, 26], [27], [28], [29], [30]. With the purpose to build advanced nanophotonic devices, it is highly desired to systematically study the optical behaviors of 2D perovskite in nanoscale. Here, we investigated nonlinear optical properties of a 2D R–P phase perovskites (BA)_2_(MA)_*n*−1_Pb_*n*_I_3*n*+1_ (*n* = 1, 2, 3, 4; BA: CH_3_(CH_2_)_3_NH_3_^+^, *n*-butyl; MA: CH_3_NH_3_^+^, methyl). The 2D perovskites are fabricated by solution cooling crystallization, and they are thin nanosheets with thickness of ∼50 nm. The nonlinear optical behaviors, including two-photon photoluminescence (2PPL), three-photon photoluminescence (3PPL) and THG, of these 2D perovskite nanosheets have been systematically studies. Based on the measurements, it is found that although these 2D perovskite nanosheets have small thickness, they exhibit large nonlinear response, for example its THG conversion efficiency is up to 8.15 × 10^−6^. Furthermore, experimental results revealed that nonlinear polarization greatly enhances the polarization dependence related to incident light, and the polarization ratio of parametric process (i.e., the THG in our case) is obviously higher than that of nonparametric process (multiphoton photoluminescence), with the largest polarization ratio of THG is as high as ∼0.99.

## 2 Results and discussions

**Figure 1:**
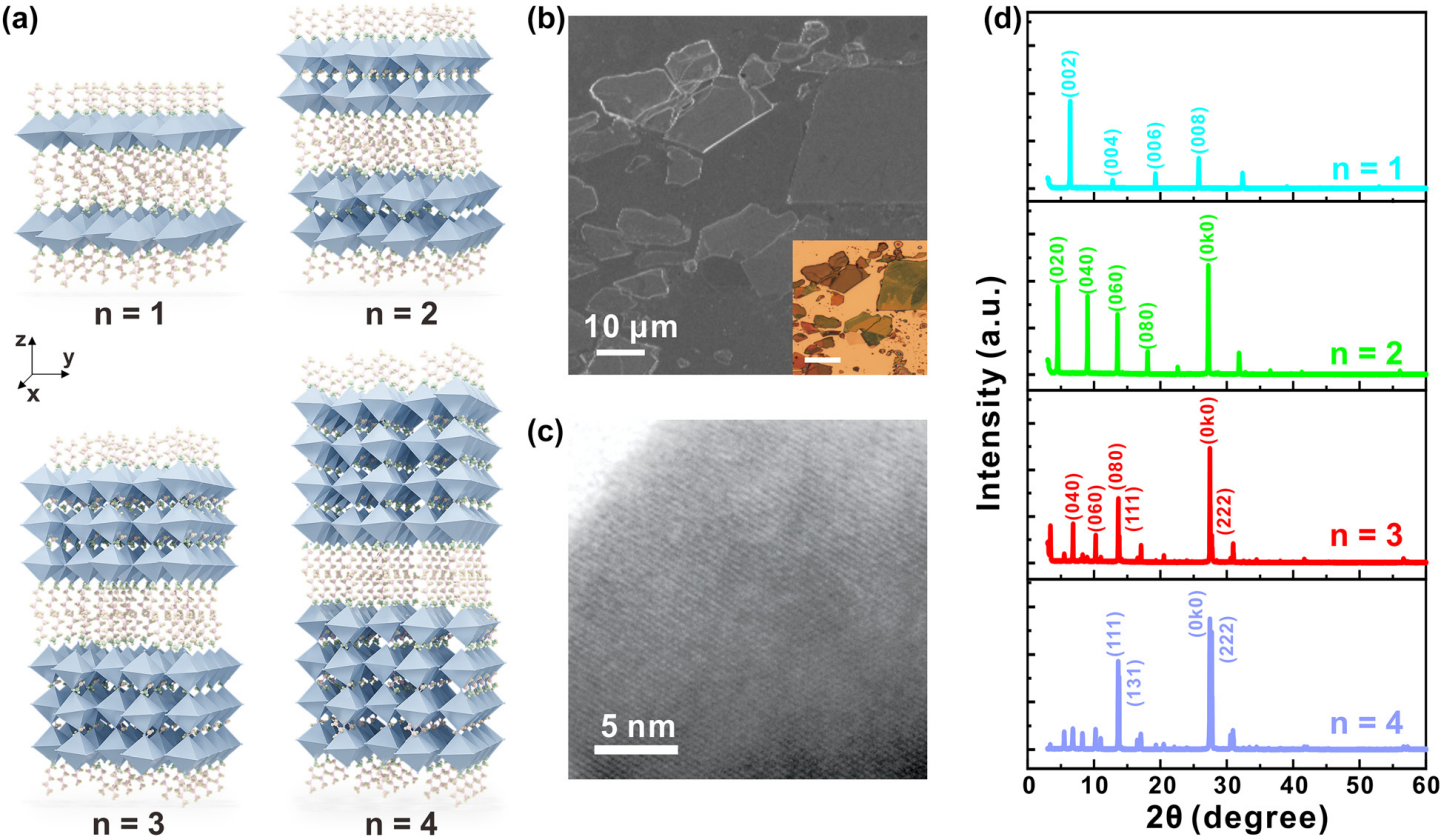
Structure and characterization of exfoliated 2D RPPs (BA)_2_(MA)_*n*−1_Pb_*n*_I_3*n*+1_(*n* = 1, 2, 3, 4) nanosheets. (a) Structure schematic diagram of 2D RPPs with different amounts of perovskites layers (*n* = 1, 2, 3, 4) sandwiched by organic spacer layers. (b) Scanning electron microscopic image of 2D RPPs (*n* = 3) nanosheets. Scale bar: 10 μm. Inset is the corresponding optical image. Scale bar: 20 μm. (c) Transmission electron microscopic image of 2D RPP (*n* = 3) nanosheets. Scale bar: 5 nm. (d) XRD patterns of the as-synthesized 2D RPP (*n* = 1, 2, 3, 4) single crystals.

The current methods for synthesizing 2D perovskites mainly include solution cooling crystallization [31], chemical vapor deposition (CVD) [32, 33], and solvent evaporation crystallization [34]. The latter two methods are used to synthesize nanoscale flakes directly, which requires harsh reaction conditions and may have a low yield. Solution cooling crystallization has been adopted widely by researchers because of simplicity and high efficiency of the synthesis process. Besides, large-scale pure phase single crystals with lateral sizes in millimeters can be obtained. Here, we obtained high quality 2D R–P phase perovskites (2D RPPs) single crystals (BA)_2_(MA)_*n*−1_Pb_*n*_I_3*n*+1_ (*n* = 1, 2, 3, 4) by solution cooling crystallization (Figure S1, Supplementary Material), which were exfoliated mechanically into nanosheets down to several layers. 2D RPPs (BA)_2_(MA)_*n*−1_Pb_*n*_I_3*n*+1_ possess Van der Waals structure (Figure 1a): [PbI_6_]^4−^ octahedrons constitute the basic inorganic framework; short-chain amine MA^+^ occupy the voids of the inorganic layer and combine with [PbI_6_]^4−^ octahedrons through ionic bonds, which form perovskite layers; long-chain amine n-BA^+^ forms spacer layers, which combine mutually by Van der Waals forces, n-BA^+^ and [PbI_6_]^4−^ octahedrons combine by ionic bonds. The weak Van der Waals forces between n-BA^+^ organic molecular spacer layers are easily destroyed under the action of external forces. Therefore, it would be facial to obtain the 2D RPP nanosheets with organic molecular layers by mechanically exfoliating.

With existence of organic composition, 2D RPPs possess lower Young’s modulus (1–10 GPa) [35] compared to graphene and transition metal dichalcogenides (TMDs, including MoS_2_, WSe_2_, etc.), which shows its intrinsic soft crystal lattice, makes it easier for mechanically exfoliating process. The abundant and tunable optical properties of 2D RPPs are mainly achieved by the diversity of organic molecules. We precisely set the ratio of reactants CH_3_NH_3_Cl and *n*-CH_3_(CH_2_)_3_NH_2_, which results in different *n* values of chemical formula (BA)_2_(MA)_*n*−1_Pb_*n*_I_3*n*+1_, corresponding to the number of perovskites layers sandwiched by organic spacers. It is difficult to obtain phase-pure samples when *n* is higher than 4 as a result of the generation of smaller *n* homologues. As *n* approaches to infinity, 2D RPPs will gradually change to their 3D counterpart which is the MAPbI_3_. Accordingly, it is vital for obtaining 2D RPPs with higher purity to fine control the molar ratio of reactants, as well as implementing reaction and crystallization in a HI environment.

The difference in lattice structures of 2D RPPs leads to the change of energy band structure. With the increase of *n* value, bandgap of 2D perovskites gradually decreases, and photoluminescence peak position is red-shifted. Figure 1b shows scanning electron microscopic (SEM) image of 2D RPPs (*n* = 3). The wavelengths of scattered light are related to the thickness of nanosheets, which is manifested by the difference in colors of samples (the inset of Figure 1b, as well as Figure S2 in Supplementary Material). The thickness of 2D RPPs nanosheets obtained by mechanically exfoliating is generally between 5 and 500 nm, with lateral size between 1 and 50 μm. Transmission electron microscopic image (Figure 1c) demonstrates the lattice arrangement of 2D RPPs, indicating that the as-synthesized single crystals have fewer defects. The X-ray diffraction patterns (Figure 1d) verifies the chemical purity of the obtained samples, which are generally consistent with theoretical calculations, indicating that 2D RPPs with phases of high purity were synthesized. From the perspective of device miniaturization and integration, the investigated 2D RPP nanosheets should be as thin as possible. However, the samples with small thicknesses (such as several layers) are not stable under the strong excitation of femtosecond laser (repetition rate = 1 kHz) in our experiments. Therefore, considering the requirements of both thin thickness and high stability under laser excitation, exfoliated 2D RPP (*n* = 1, 2, 3, 4) nanosheets with thickness of about 50 nm were used, and the thicknesses were confirmed by atomic force microscope (AFM) (Figure S3, Supplementary Material).

Light absorption is the most basic form of interactions between light and matter, including single-photon absorption and multi-photon absorption. For two-level semiconductor materials, multiphoton absorption becomes mainstream on conditions that the excitation photon energy *E*_p_ is less than the band gap *E*_g_, as well as the intensity of incident laser is strong enough. As direct band gap semiconductor materials with tunable bandgaps, 2D RPPs possess significant multi-photon absorption (two/three photons absorption) under the excitation of femtosecond laser. The structure of 2D RPPs with organic spacer layers (*n*-BA^+^) and perovskite layers arranging alternatively, forms vertical type-I MQWs, in which the organic spacer layers function as dielectric barrier layers [17]. When 2D RPPs single crystals gradually become two-dimensional (∼50 nm) through the process of mechanical exfoliate, their quantum and dielectric confinement effects will be greatly enhanced, bringing about electron-hole pairs localized in the perovskite layers, known as free excitons.

**Figure 2:**
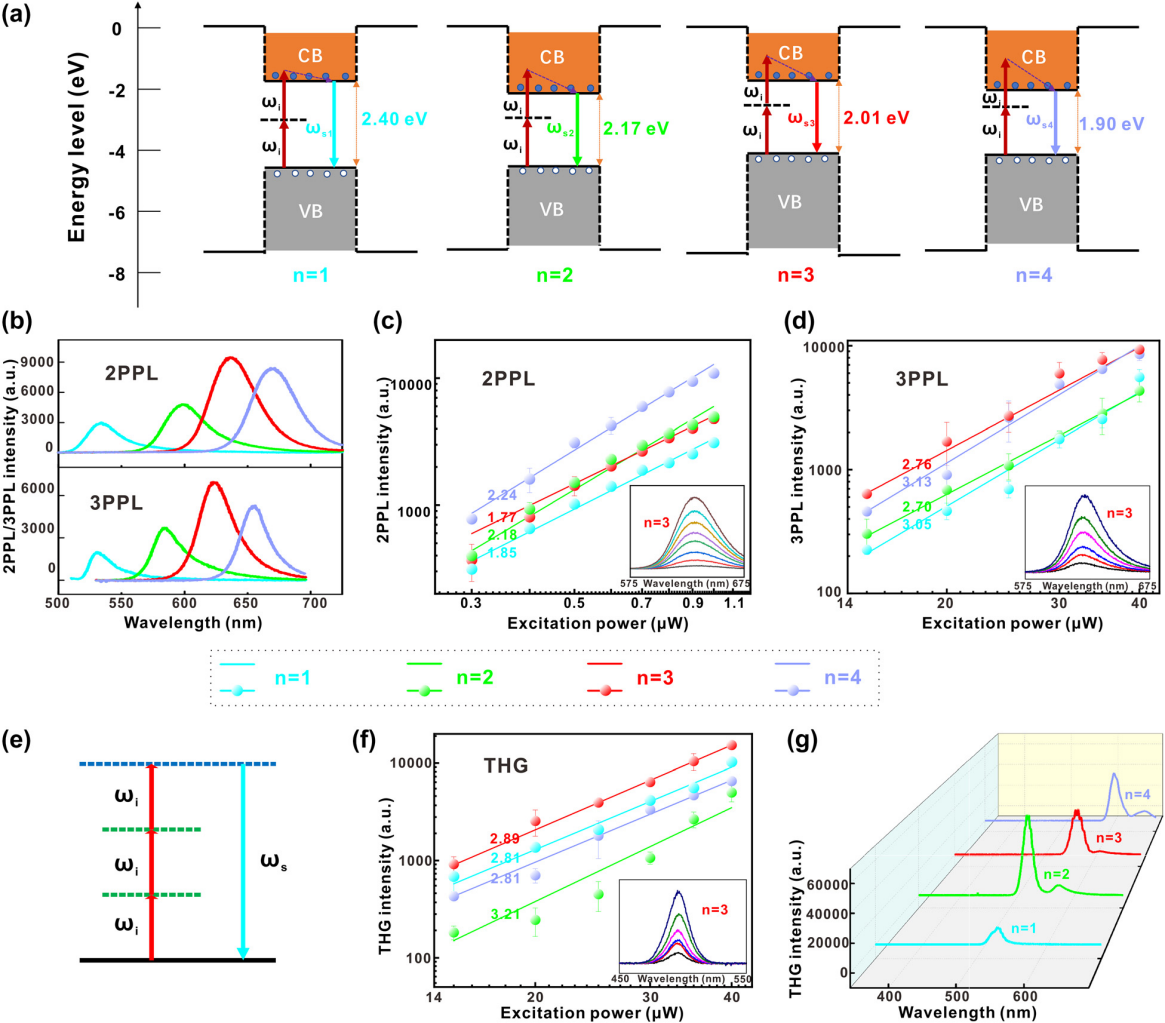
Multi-photon photoluminescence and THG responses of the 2D RPPs (BA)_2_(MA)_*n*−1_Pb_*n*_I_3*n*+1_ (*n* = 1, 2, 3, 4) nanosheets. (a) Energy band schematic diagrams of two photons absorption in single quantum well of 2D RPP (*n* = 1, 2, 3, 4) nanosheets. (b) Typical 2PPL/3PPL spectra of different 2D RPPs (*n* = 1, 2, 3, 4). Excitation powers are 1 and 30 μW for 2PPL and 3PPL, respectively. The excitation wavelengths are 800 and 1500 nm for the 2PPL and 3PPL, respectively. (c) and (d) Peak intensities of 2PPL/3PPL in 2D RPP (*n* = 1, 2, 3, 4) nanosheets under different excitation powers. Linear fittings are carried out on the double logarithmic coordinate system. The insets show 2PPL/3PPL spectra of 2D RPPs (*n* = 3) under different excitation powers (0.3–1.0 μW, step = 0.1 μW for 2PPL; 15–40 μW, step = 5 μW for 3PPL). (e) Energy band schematic diagram of THG, among them *ω*_i_/*ω*_s_ is circular frequency of excitation/signal photons. (f) Peak intensities of THG versus excitation powers, fitted linearly in double logarithmic coordinate system. The excitation wavelength is 1500 nm. The inset is the evolution of THG spectra from the 2D RPPs (*n* = 3) under different excitation powers (15–40 μW, step = 5 μW). (g) The spectra of 2D RPPs (*n* = 1, 2, 3, 4) when they reached the largest *η*_THG_. The excitation power is 40 μW. The excitation wavelengths are 1600, 1700, 1750, and 1800 nm for the 2D RPPs with *n* = 1, 2, 3, and 4, respectively.

Here, 2D RPPs (*n* = 1, 2, 3, 4) nanosheets have been delicately modulated with optical bandgaps of 2.4 eV, 2.17 eV, 2.01 eV, and 1.90 eV, respectively (demonstrated by the photoluminescence spectra in Figure 2b, without considering Stokes shifts). Figure 2a shows the generation processes of two-photon absorption induced fluorescence in 2D RPP (*n* = 1, 2, 3, and 4) nanosheets: under the excitation of the 800 nm fs laser, a valence band electron in the quantum well first absorb a photon to transition to virtual energy level, while leaving one hole in the valence band, and then absorbs a photon to transition to conduction band; the conduction band electrons in excited state transition back to valence band through radiation, with radiated photons of energies approximately equal to the bandgaps, which is 2PPL. The generation process of 3PPL is basically similar to 2PPL, except that the excitation wavelength is changed to 1500 nm, and the valence band electrons need to absorb three photons at the same time, passing through two virtual energy levels.

It is worth noting that due to the short lifetime of virtual energy levels (levels of femtosecond), it is usually a femtosecond pulse laser with strong peak power that can ensure the completion of excitons transition process in such a short time, thereby strong nonlinear optical effects can be observed. High-purity 2D RPP (*n* = 1, 2, 3, and 4) nanosheets produce 2PPL and 3PPL (Figure 2b) of excellent monochromaticity with full width at half maximum about 30 nm. The peak wavelengths are around 525 nm, 590 nm, 627 nm and 656 nm, influenced by thickness of nanosheets (variation range ∼ 20 nm). In Figure 2c and d, the peak intensities of 2PPL/3PPL versus excitation powers of incident laser is fitted linearly in double logarithmic coordinate system. As a result, slopes of the obtained curves are around 2.0/3.0 for 2PPL/3PPL photoluminescence. According to the relationship: $I_{2\text{PPL}}\propto {I_{\text{exc}}}^{2}$ and $I_{3\text{PPL}}\propto {I_{\text{exc}}}^{3}$ , the generation of 2PPL and 3PPL can be confirmed.

The above-mentioned multiphoton absorptions are non-parametric processes in which energy exchange occurs between light and medium, classified as active nonlinear optical effect. Besides, there are parametric processes in which energy exchange merely occurs between light and light, belonging to passive nonlinear optical effect, such as SHG, THG, four waves mixing, etc. There is also strong THG in 2D RPPs semiconductor materials with MQWs structure, which will be applied widely in field of all-optical modulators [19]. Although theoretically THG exists in varieties of materials, it can seldom be observed by researchers in most crystals, for the reason that the third-order polarizability of general nonlinear medium is small, and the laser damage threshold is not high as well, resulting in no obvious THG under the limited excitation power.

Figure 2e shows energy levels schematic diagram of THG. According to conservation of energy, there is a relationship: *ω*_*i*_ + *ω*_*i*_ + *ω*_*i*_ = *ω*_*s*_, with corresponding wavelengths relationship: *λ*_*s*_ = 1/3*λ*_*i*_. As a parametric process, the energy levels diagrams of THG are basically similar in different 2D RPPs (*n* = 1, 2, 3, 4). We choose 1500 nm for excitation with the purpose of independently investigating the spectral evolution of THG under different excitation powers (Figure 2f). In Figure 2f we fit linearly peak intensities of THG versus excitation powers in double logarithmic coordinate system and the slopes of obtained curves are all around 3.0. Therefore, the generation of the third harmonic can be determined according to the relationship: $I_{\text{THG}}\propto {I_{\text{exc}}}^{3}$ .

We calculate the THG conversion efficiency *η*_THG_ by the equations as follow:

$$\frac{\bar{P_{\text{s}}}\times B}{I_{\text{s}}}=\frac{\bar{P_{0}^{\text{R}}}\times A\times B}{I_{\text{R}}}$$

$$P_{\text{in}}=A\bar{P_{0}}/\nu \tau$$

$$P_{\text{s}}=\bar{P_{\text{s}}}/\nu \tau$$

$$\eta_{\text{THG}}=P_{\text{s}}/P_{\text{in}}$$

where $\bar{P_{\text{s}}}$ is the average power of signal light (for example, emission with wavelength of 500 nm for THG), $I_{\text{s}}$ is the intensity of signal light which is obtained by a spectrometer. *A* (*A* = 50%) and *B* are the losses of excitation and collecting systems, respectively. A reference laser was applied in our calculations, and its average power was measured as $\bar{P_{0}^{\text{R}}}$ , with its intensity being recorded as the integration counts of *I*_R_. Besides, $\bar{P_{\text{0}}}$ is the average power of excitation laser. *P*_in_ and *P*_s_ are power of the incident light and THG signal, respectively. The laser repetition rate *ν* is 1 kHz, and the laser pulse length *τ* is 33 fs. Also, in order to obtain systematical results, the excitation wavelengths have been varied. Based on these conditions, the largest *η*_THG_ of 2D RPP nanosheets are calculated as 2.95 × 10^−6^, 6.40 × 10^−6^, 8.15 × 10^−6^, 7.35 × 10^−6^ for *n* = 1, 2, 3, 4, respectively. The original THG spectra are given in Figure 2g, and more data about the THG spectra and corresponding *η*_THG_ of 2D RPPs (*n* = 1, 2, 3, 4) under different excitation wavelengths are shown in Figure S4 (Supplementary Material). Table S1 in Supplementary Material is a comparison of THG conversion efficiency for different materials. Based on the results shown in Table S1, one can find that the efficiencies of our 2D perovskite nanosheets are impressive.

**Figure 3:**
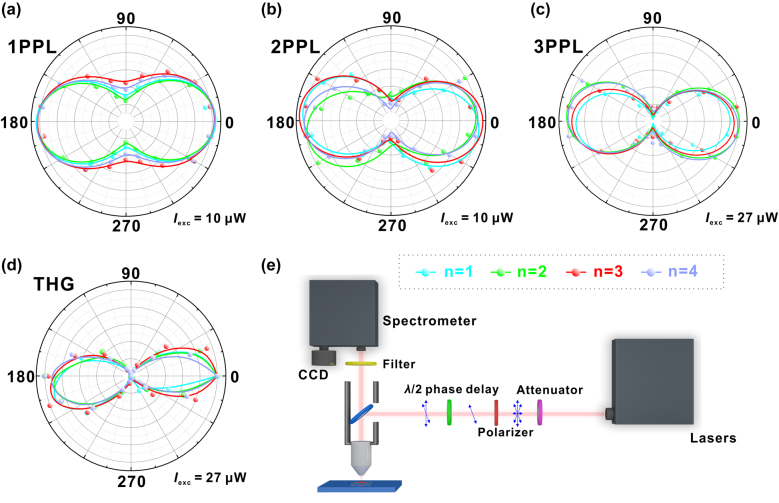
Incident light polarization dependence of 2D RPPs’ different optical processes. (a) Incident light polarization dependence of single-photon photoluminescence (1PPL). Excitation wavelength is 400 nm. (b) Incident light polarization dependence of 2PPL. Excitation wavelength is 800 nm. (c) Incident light polarization dependence of 3PPL. Excitation wavelength is 1500 nm. (d) Incident light polarization dependence of THG. Excitation wavelength is 1800 nm. (e) Schematic diagram of polarization dependence measurement setup.

Large optical anisotropy has been especially observed in the high-order nonlinear processes of 2D RPP nanosheets (*n* = 1, 2, 3, 4). Figure 3a–d are the linear polarization dependence of 1PPL/2PPL/3PPL, and THG versus polarized angles of incident light, among which Figure 3a functions as a control group for linear excitation. The typical spectra of 2D RPPs (*n* = 3) in the polarization dependence measurements are shown in Figure S5 (Supplementary Material). Judging from the shape of polarization dependence curves, there are similar tendencies for 1PPL, 2PPL/3PPL, and THG, all with a shape of “two lobes”. The unique lattice structures of 2D RPPs (*n* = 1, 2, 3, 4) can be the reason for the two-lobe shape of polarization curves (Figure S6) [36]. In addition, the anisotropic excitons generated by the different physical properties of organic spacers and perovskite octahedrons have direct impact on the optical polarization dependence of 2D RPPs [30]. Furthermore, the carrier dynamics of 2D perovskite are highly dependent on the composition of inorganic and organic part [37], which should also play an important role for the nonlinear optics. The number of perovskite layers (*n* value) has basically no effect on anisotropy, because anisotropic bright excitons in in-plane quantum wells can hardly be affected by the vertical lattice anisotropy.

Although nonlinear excitation does not change the shape of linear polarization dependence curves of 2D RPPs, it has significant impact on polarization ratio of the linear polarization dependence. It can be observed from Figure 3a–d that the “gap” at the valleys of 1PPL polarization dependence curves is large, which is significantly reduced for 2PPL and even disappears for 3PPL and THG. The phenomena show that the optical anisotropy of 2D RPPs has been significantly enhanced under nonlinear excitation. Polarization ratio *P* is calculated according to the following formulas:

$$P=\frac{I_{\text{max}}-I_{\text{min}}}{I_{\text{max}}+I_{\text{min}}}$$

Among them, *I*_max_ and *I*_min_ are the maximum and minimum values of signal light in a period (180°), respectively. Accordingly, we can calculate the *P* values, and the results of the THG process are 0.986, 0.962, 0.955, and 0.975 for the 2D RPPs with *n* = 1, 2, 3, 4, respectively. Also, our experimental setup is shown in Figure 3e. A wide-band tunable femtosecond laser is used for excitation. The laser intensity is reduced to an appropriate level through an attenuator, and then the partially polarized light is transformed into a completely linearly polarized light through a linear polarizer. By rotating the *λ*/2 phase retarder with an angle of *φ* (0° ≤ *φ* < 180°, *θ* = 2*φ*), linearly polarized light in all directions in-plane is obtained, which is focused on the samples by microscope for excitation. Finally, the signal light is collected by a visible spectrometer.

**Figure 4:**
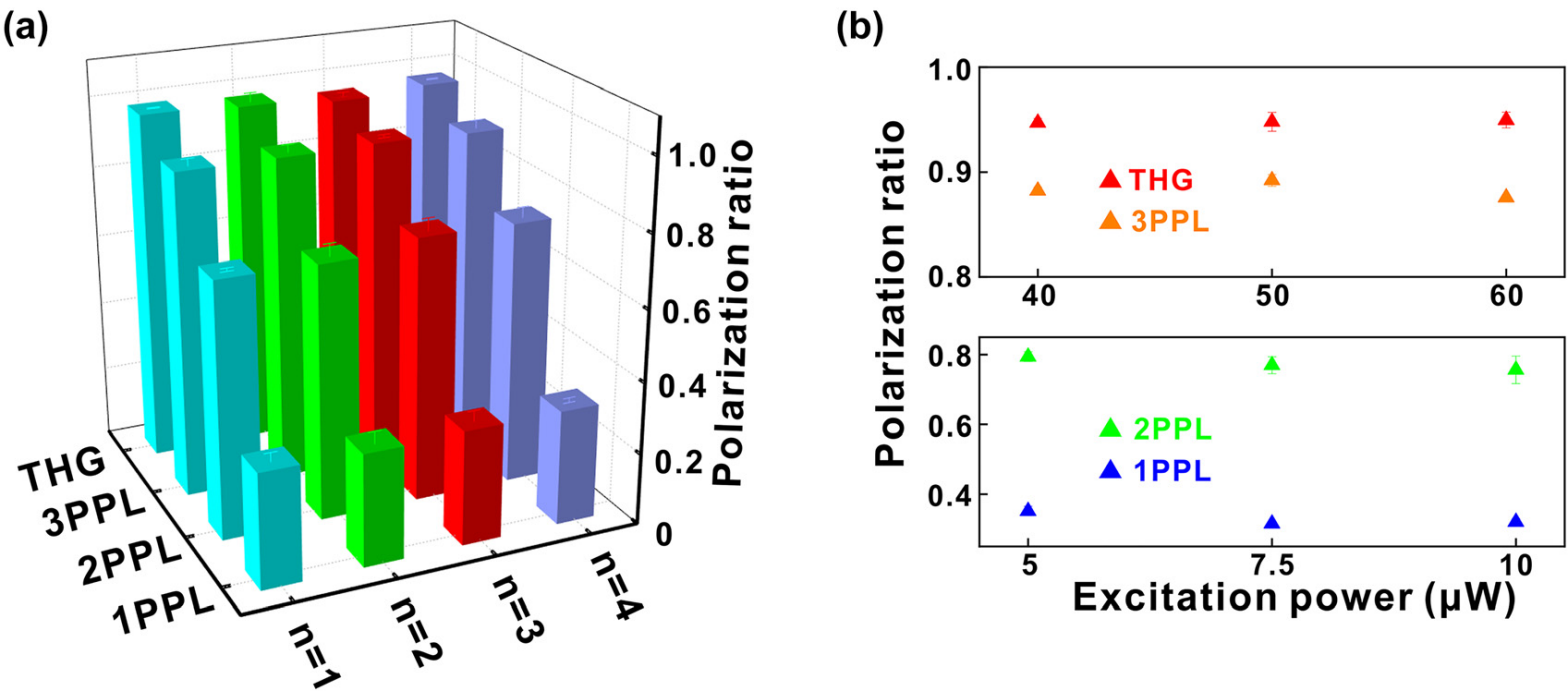
Comparison of polarization ratios of 2D RPPs linear polarization dependence for different optical processes. (a) Polarization ratios of 1PPL/2PPL/3PPL and THG for 2D RPPs linear polarization dependence. The excitation powers are 10 μW for the 1PPL and 2PPL processes, and are 27 μW for the 3PPL and THG processes. The excitation wavelengths are 400, 800, 1500, and 1800 nm for the 1PPL, 2PPL 3PPL, and THG processes, respectively. (b) Polarization ratios of 1PPL/2PPL/3PPL and THG for 2D RPPs (*n* = 2) linear polarization dependence under different excitation powers.

Figure 4a is a comparison of the polarization ratios of 2D RPPs (*n* = 1, 2, 3, 4) for different optical processes. It can be clearly seen that for nonparametric processes, the polarization ratios under nonlinear excitation (2PPL/3PPL) are remarkably higher than that under linear excitation (1PPL), and the polarization ratios of 3PPL is higher than that of 2PPL; the polarization ratio of THG is higher than that of 3PPL. The highest polarization ratio is obtained based on the THG process with a value of 0.991 (*n* = 2), which is significantly higher than that obtained in previous related literature (Table S2, Supplementary Material). Such large optical anisotropy can also be attributed to the reason that, the intrinsic soft lattice structure of 2D RPPs makes it easier to generate dynamic disorder and distortion caused by the vibrations of perovskite sublattices under the laser excitation, leading to the generation of anisotropic free excitons, which largely enhance the optical anisotropy of 2D RPPs [38].

In order to avoid the influence of excitation powers on the experimental results and confirm the enhancement effect of nonlinear excitation on the optical anisotropy of 2D RPPs, different excitation powers were applied to investigate the linear polarization dependence of 2D RPPs (*n* = 2). The conclusions above can be confirmed due to rather little change of polarization ratios in different optical processes (Figure 4b). It should be mentioned that due to the excitation manner and the thin thickness of our 2D RPP nanosheets, we only investigated the in-plane optical anisotropy of our samples.

Large optical anisotropy is a quite important property for the design of polarization-sensitive optoelectronic devices, greatly benefiting for the fields of polarized light detection, imaging and optical communication [39], [40], [41]. Usually, the miniaturized and integrated polarization-sensitive optoelectronic devices are built by 2D layered materials such as black phosphorus [42], compound of Group IV–V [43, 44] and Group IV–VI [45], organic semiconductors [46], transition metal dichalcogenides [47], [48], [49], van der Waals heterojunction [50, 51]. But, these materials still have disadvantages, e.g., instability in environmental conditions, narrow optical response range, and insufficient anisotropic photocurrent rate [39]. Therefore, our findings undoubtedly present a new nanomaterial system for making polarization-sensitive nanodevices.

## 3 Conclusions

In summary, we synthesized 2D RPPs (*n* = 1, 2, 3, 4) by solution cooling crystallization, and obtained nanosheets with thickness of about 50 nm by mechanical exfoliation. We systematically investigated the nonlinear optical properties of 2D RPPs (*n* = 1, 2, 3, 4) nanosheets, including 2PPL, 3PPL, and THG. Strong conversion efficiency of THG with the highest value up to ∼8 × 10^−6^ is obtained, which is due to the large quantum and dielectric confinement in self-assembled MQWs. Furthermore, we have found that nonlinear polarization has great enhancement on the polarization ratios of incident polarization dependence, mainly as follows: the higher nonlinear order of multi-photon photoluminescence, the greater polarization ratios obtained; the polarization ratios of THG are greater than that of multi-photon photoluminescence. We obtain the highest polarization ratio up to ∼0.99 in 2D RPPs for THG process. Our research on nonlinear optics of 2D RPPs not only enriches the understandings of nonlinear polarized optical properties of 2D perovskites, but also paves the way for future research on nonlinear polarized integrated photonic devices.

## 4 Methods

### 4.1 Samples preparation

#### Synthesis of (BA)_2_(MA)_*n*−1_Pb_*n*_I_3*n*+1_ (*n* = 1, 2, 3, 4)

The yellow PbO powders (1116 mg) are dissolved in a mixture of HI (57% aqueous solution, 5 mL) and H_3_PO_2_ (50% aqueous solution, 850 μL), and hot PbI_2_ solution was obtained by magnetic stirring at 110 °C in an oil bath for 5 min, to which solid CH_3_NH_3_Cl (*x* mg) is added in order to obtain CH_3_NH_3_PbI_3_ solution (*x* = 0, 169, 225, 254 for synthesizing the 2D RPPs with *n* = 1, 2, 3, 4, respectively). In a 100 mL beaker, HI (2.5 mL) and *n*-CH_3_(CH_2_)_3_NH_2_ (*m* μL) are neutralized in an ice bath to obtain *n*-CH_3_(CH_2_)_3_NH_3_I solution (*m* = 462, 347, 164, 124 for *n* = 1, 2, 3, 4, respectively). The n-CH_3_(CH_2_)_3_NH_3_I solution is added to the PbI_2_ solution, which is stirred magnetically for 2 min in an oil bath at 110 °C. The stirring is stopped and the solution is cooled to room temperature. After 2 h, orange–yellow/dark-purple/brown–black/dark-black crystals (for *n* = 1, 2, 3, 4, respectively) are obtained by filtering with suction, which are dried in an oven at 40 °C for 24 h. Finally, we obtain 2D nanosheets by mechanically exfoliating from the crystal samples. The schematic diagram of sample preparation can also be found in Figure S1, Supplementary Material.

### 4.2 Material characterizations

Scanning electron microscopic images are obtained by a scanning electron microscope (ZEISS AURIGA). Optical images are taken by an optical microscope (OLYMPUS BX53). The PL and THG measurements are implemented on a visible spectrometer (Princeton Instruments SP-2500). Femtosecond laser originates from an integrated Ti:Sapphire femtosecond laser amplifier (Astrella-Tunable-USP-1K) and an integrated femtosecond parametric amplifier (COHERENT OPERA-SOLO). X-ray diffraction patterns are obtained by a polycrystalline X-ray diffractometer (Empyrean). Transmission electron microscopic images are taken by a 200 kV transmission electron microscopy (JEM-2010HR). Thickness of samples are obtained by an atomic force microscope (Dimension Icon).

## Supplementary Material

Supplementary Material Details
