# Supplementary material for: High efficiency and large optical anisotropy in the high-order nonlinear processes of 2D perovskite nanosheets
Source: Nanophotonics. 2022 Mar 1;11(7):1379–87. doi: 10.1515/nanoph-2021-0789 (PMC11501272; doi:10.1515/nanoph-2021-0789)
Supplement: Supplementary file 1 — Supplementary Material Details [file j_nanoph-2021-0789_suppl.pdf]

Supplementary Material for

High efficiency and large optical anisotropy in the high-order nonlinear processes of 2D perovskite nanosheets

Zehong Chen[1,#], Zhonghong Shi[1,#], Wenbo Zhang[1], Zixian Li[1], Zhang-Kai Zhou[1,*]

[1]*State Key Laboratory of Optoelectronic Materials and Technologies, School of Physics, Sun Yat-sen University, Guangzhou 510275, China*

[#]*These authors contributed equally*

**Corresponding Author: zhouzhk@mail.sysu.edu.cn*

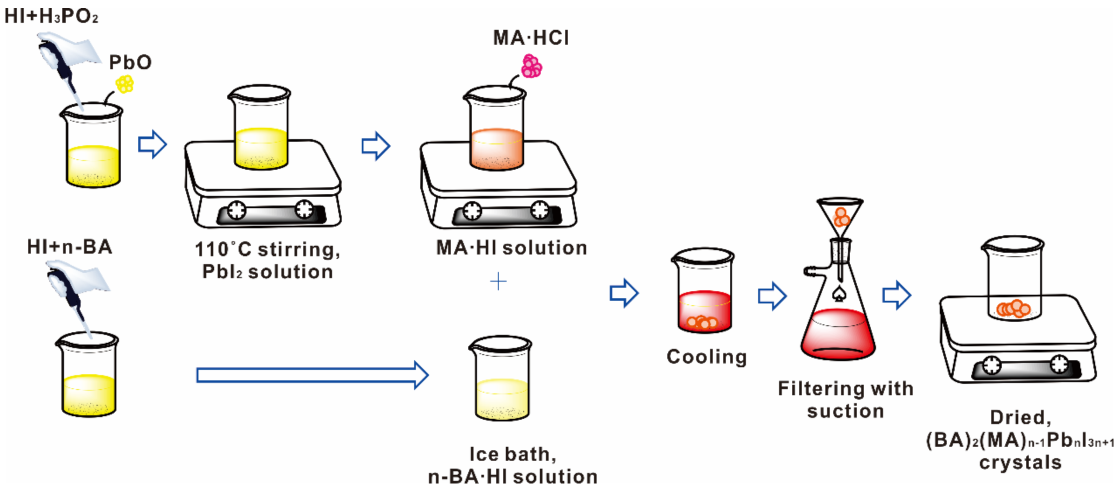

Fig. S1 The schematic diagram of samples preparation.

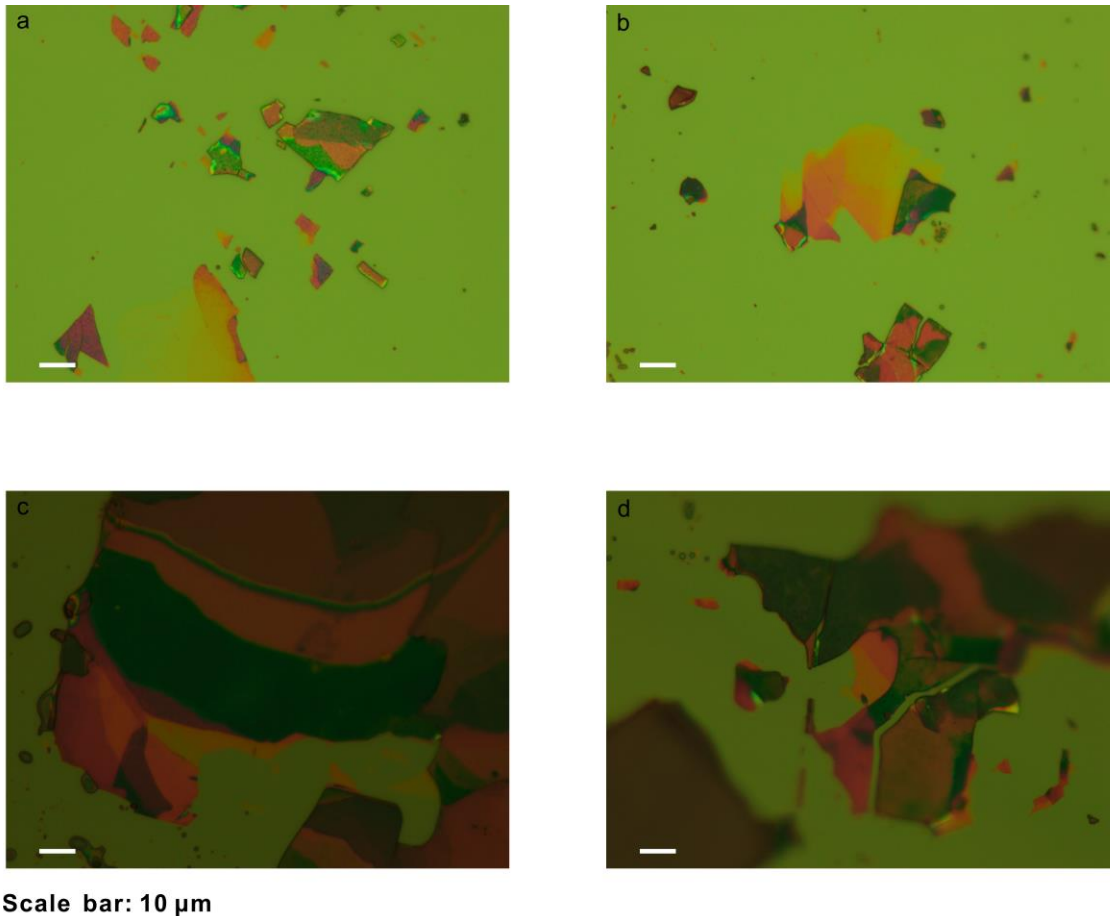

Fig. S2 Optical images of 2D RPPs (n = 1, 2, 3, 4)**.** (a)-(d) are optical images of n = 1, 2, 3, 4, respectively. Scale bar is 10 μm.

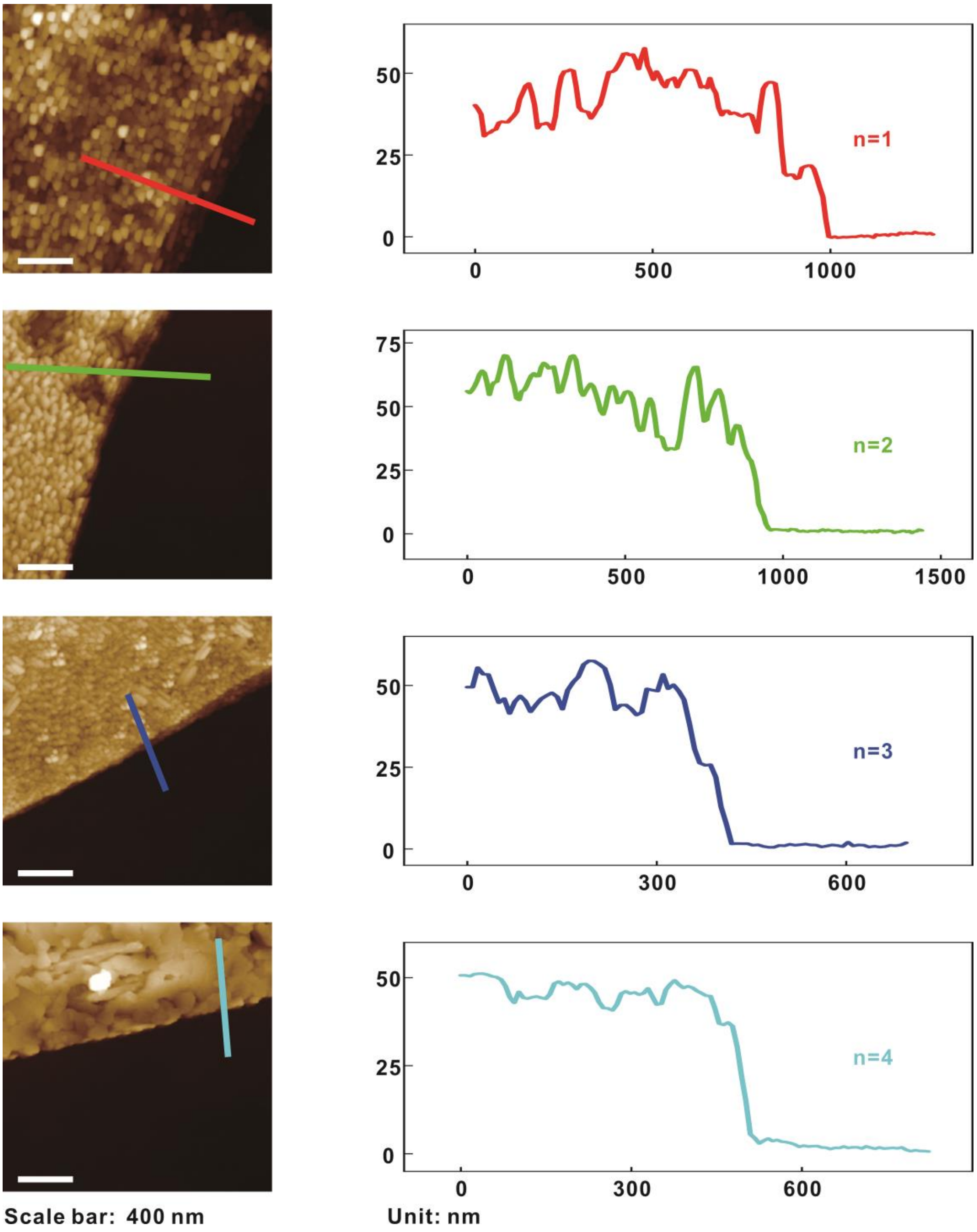

Fig. S3 Atomic force microscopic images of 2D RPPs（n = 1, 2, 3, 4）. Figures from top to bottom are AFM measurements of n = 1, 2, 3, 4, respectively. Their thicknesses are measured all around 50 nm. Scale bar is 400 nm.

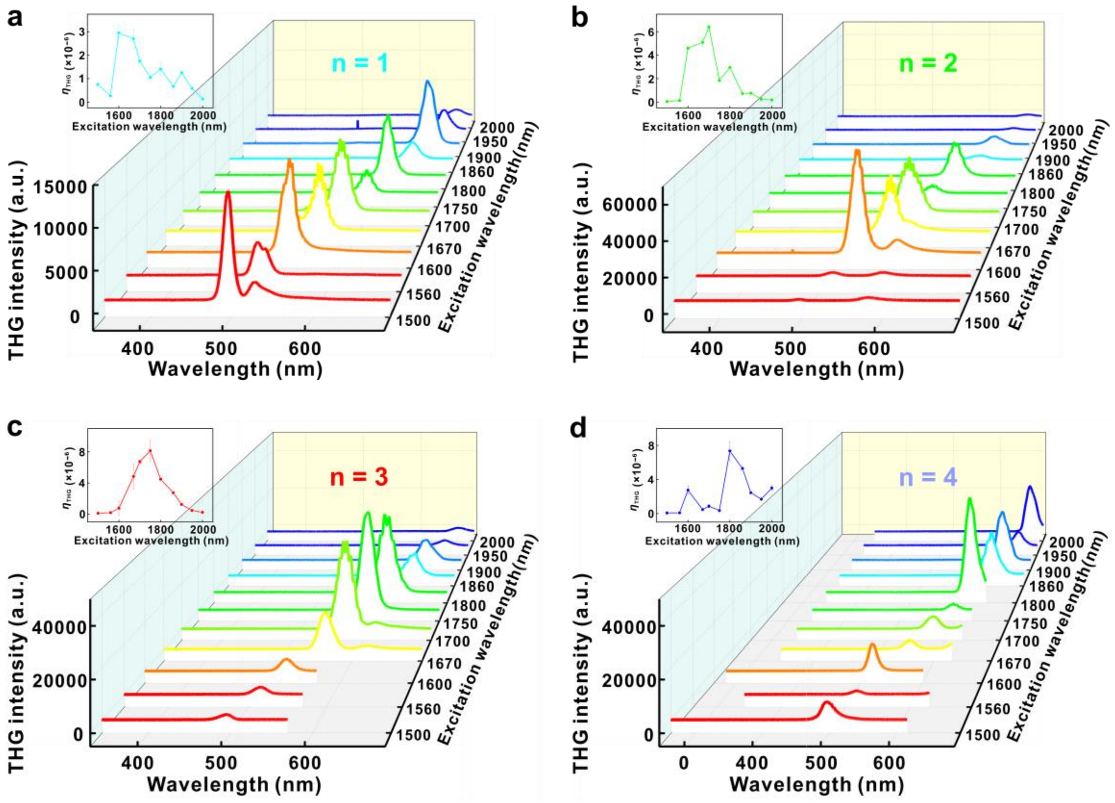

Fig. S4 THG spectra and their corresponding efficiencies of 2D RPPs (n = 1, 2, 3, 4) under different excitation wavelengths. The excitation power is 40 μW.

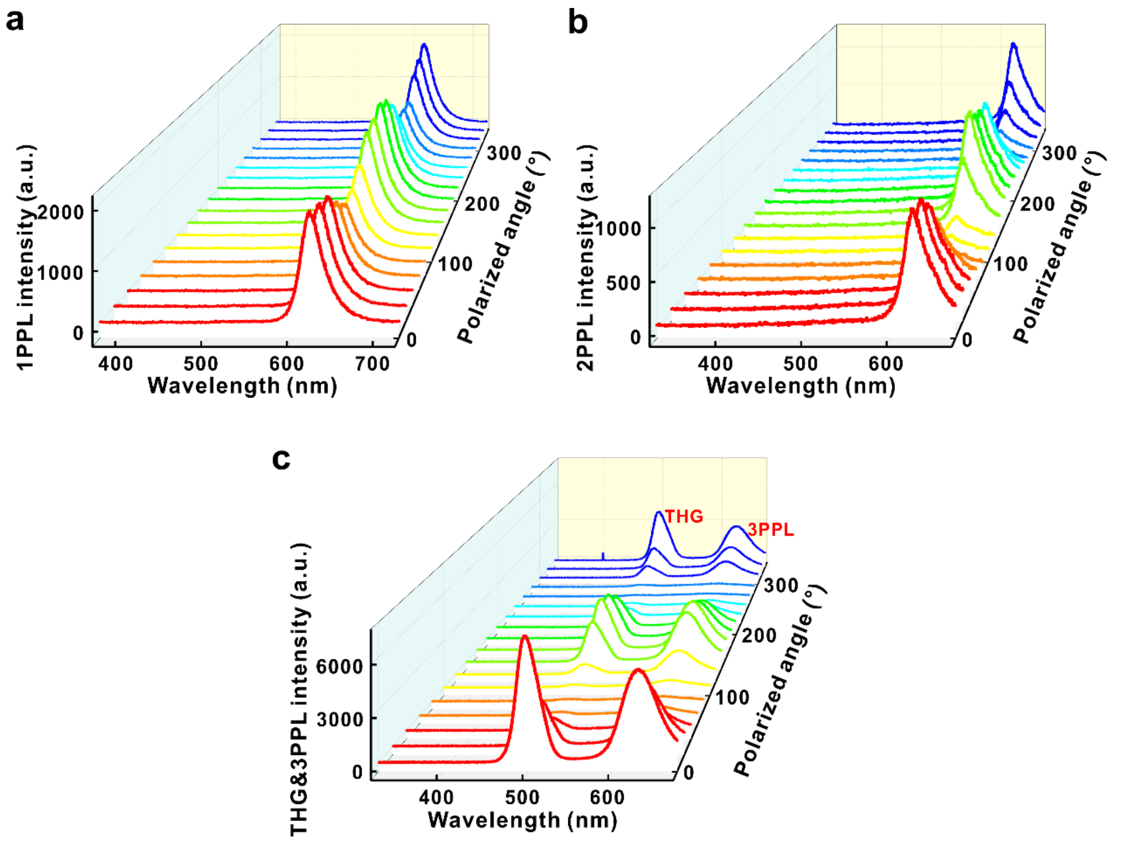

Fig. S5 Polarization dependence spectra of 1PPL(a), 2PPL(b), 3PPL and THG(c) for 2D RPPs (n = 3) nanosheets. The excitation wavelengths are 400 (a), 800 (b), and 1500 (c) nm, respectively.

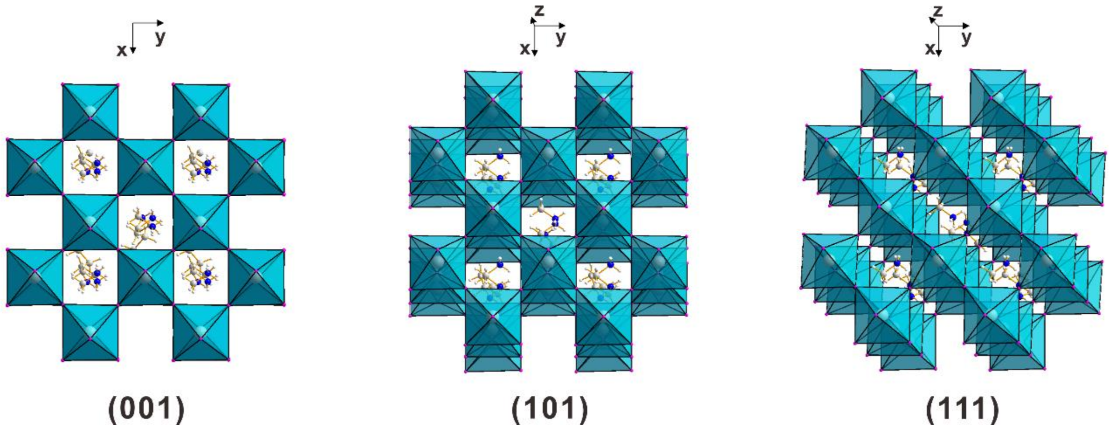

Fig. S6 The schematic diagrams of lattice structure of $(BA)_2(MA)_2Pb_3I_{10}$ (n = 3) from different views.

Table S1 Comparison of THG conversion efficiency for different materials.

**Materials**	**Structure**	**Emission wavelength**	**Conversion efficiency**	**Ref.**
$(BA)_2(MA)_{n-1}Pb_nI_{3n+1}$(n = 1, 2, 3); $(BA)_2PbBr_4$	90 nm nanosheets encapsulated with h-BN	558 nm	6.0×10^{-5}	[1]
Germanium	200 nm nanodisk	550 nm	10^{-5}	[2]
MoS_2	Few layers	520 nm	4.76×10^{-10}	[3]
Silicon	Nanodisk arrays	413 nm	10^{-7}	[4]
Silicon	Quadrumers of silicon nanodisks	447 nm	10^{-6}	[5]
Silicon	Asymmetric metasurfaces	480 nm	10^{-6}	[6]
Au/SiO_2	Multilayered metal–dielectric nanocavities	520 nm	1.8×10^{-10}	[7]
Perovskites crystals $CH(NH_2)_2PbBr_3$	300 nm films	513 nm	4.27×10^{-6}	[8]
Silicon	Microspherical Resonators	520 nm	10^{-5}	[9]
Silicon/Al_2O_3	Metasurfaces	1.12 μm	10^{-9}	[10]
$(BA)_2(MA)_{n-1}Pb_nI_{3n+1}$ (n = 1, 2, 3, 4)	50 nm nanosheets	1600 - 1800 nm	$3-8\times10^{-6}$	This work

Discussion. Based on the Table S1, one can see that the THG conversion efficiencies ranging from 3×10^{-6} to 8×10^{-6} obtained in our 2D RPPs are impressive results, especially with the consideration of the results obtained in thick perovskite films [8] and few layered MoS_2 [3]. In addition, our results are also comparable to nanostructures with fine resonant designs, which the THG efficiency has been enhanced.

Table S2 Comparison of linearly polarized dependence polarization ratio for different perovskites.

Materials	Morphology	Optical process	Max polarization ratio	Ref.
$(C_6H_5(CH_2)_2NH_3)_2PbI_4$; $(C_6H_{11}NH_3)_2PbI_4$; $(C_4H_9NH_3)_2PbI_4$	Bulk	THG; 2PPL; 3PPL	0.946; 0.6; 0.655	[11]
$(R\text{-}MPEA)_{1.5}PbBr_{3.5}(DMSO)_{0.5}$; $(S\text{-}MPEA)_{1.5}PbBr_{3.5}(DMSO)_{0.5}$	Nanowire	SHG	0.964	[12]
R-; *S*- and rac-$ClPEA_2PbI_4$	Microwire arrays	SHG; 2PPL	0.917	[13]
$(IA)_2(EA)_2Pb_3Br_{10}$	Bulk	SHG	0.846	[14]
$[(C_6H_5CH_2NH_3)_2]PbCl_4$	Nanosheets	SHG	0.62	[15]
$MAPbI_3$	Mono crystal	1PPL	0.70	[16]
$CsPbBr_3$	Ultrathin nanowires	1PPL	0.78	[17]
$(BA)_2PbI_4$	Nanowires	1PPL	0.73	[18]
$CsPbBr_3$/polymers	Aligned nanowires film	1PPL	0.44	[19]
$CsPbBr_3$/polymer matrix	Aligned NRs-PM films	1PPL	0.23	[20]
$(BA)_2(MA)_{n-1}Pb_nI_{3n+1}$ (n = 1, 2, 3, 4)	Nanosheets	THG; 3PPL; 2PPL	0.99; 0.92; 0.76	This work

Reference

[1] Abdelwahab I, Grinblat G, Leng K, et al. Highly Enhanced Third-Harmonic Generation in 2D Perovskites at Excitonic Resonances. ACS Nano 2018;12:644-650.

[2] Grinblat G, Li Y, Nielsen MP , Oulton RF, & Maier SA. Efficient Third Harmonic Generation and Nonlinear Subwavelength Imaging at a Higher-Order Anapole Mode in a Single Germanium Nanodisk. ACS Nano 2017;11:953-960.

[3] Säynätjoki A, Karvonen L, Rostami H, et al. Ultra-strong nonlinear optical processes and trigonal warping in MoS_2 layers. Nat Commun 2017;8:893.

[4] Shcherbakov MR, Neshev DN, Hopkins B, et al. Enhanced Third-Harmonic Generation in Silicon Nanoparticles Driven by Magnetic Response. Nano Lett 2014;14:6488-6492.

[5] Shorokhov AS, Melik-Gaykazyan EV, Smirnova DA, et al. Multifold Enhancement of Third-Harmonic Generation in Dielectric Nanoparticles Driven by Magnetic Fano Resonances. Nano Lett 2016;16:4857-4861.

[6] Koshelev K, Tang YT, Li KF, et al. Nonlinear Metasurfaces Governed by Bound States in the Continuum. ACS Photon 2019;6:1639-1644.

[7] Maccaferri N, Zilli A, Isoniemi T, et al. Enhanced Nonlinear Emission from Single Multilayered Metal–Dielectric Nanocavities Resonating in the Near-Infrared. ACS Photon 2021;8:512-520.

[8] Rubino A, Huq T, Dranczewski JK, et al. Efficient third harmonic generation from $FAPbBr_3$ perovskite nanocrystals. J Mater Chem C 2020;8:15990-15995.

[9] Farnesi D, Barucci A, Righini GC, et al. Optical Frequency Conversion in Silica-Whispering-Gallery-Mode Microspherical Resonators. Phys Rev Lett 2014;112:093901.

[10] Shcherbakov MR, Werner K, Fan ZY, et al. Photon acceleration and tunable broadband harmonics generation in nonlinear time-dependent metasurfaces. Nat Commun 2019;10:1345.

[11] Chen Z, Zhang Q, Zhu ML, et al. In-Plane Anisotropic Nonlinear Optical Properties of Two-Dimensional Organic-Inorganic Hybrid Perovskite. J Phys Chem Lett 2021;12;7010-7018.

[12] Yuan CQ, Li XY, Semin S, et al. Chiral Lead Halide Perovskite Nanowires for Second-Order Nonlinear Optics. Nano Lett 2018;18:5411-5417.

[13] Zhao JJ, Zhao YJ, Guo YW, et al. Layered Metal-Halide Perovskite Single-Crystalline Microwire Arrays for Anisotropic Nonlinear Optics. Adv Funct Mater 2021;31(48):2105855.

[14] Ma Y, Wang JQ, Guo WQ, et al. The First Improper Ferroelectric of 2D Multilayered Hybrid Perovskite Enabling Strong Tunable Polarization-Directed Second Harmonic Generation Effect. Adv Funct Mater 2021;31:2103012.

[15] Wei WJ, Jiang XX, Dong LY, et al. Regulating Second-Harmonic Generation by van der Waals Interactions in Two-dimensional Lead Halide Perovskite Nanosheets. J Am Chem Soc 2019;141:9134-9139.

[16] Täuber D, Dobrovolsky A, Camacho R & Scheblykin IG. Exploring the Electronic Band Structure of Organometal Halide Perovskite via Photoluminescence Anisotropy of Individual Nanocrystals. Nano Lett 2016;16:5087-5094.

[17] Gao Y, Zhao LY, Shang QY, et al. Ultrathin $CsPbX_3$ Nanowire Arrays with Strong Emission Anisotropy. Adv Mater 2018;30:1801805.

[18] Ghoshal D, Wang TM, Tsai HZ, et al. Catalyst-Free and Morphology-Controlled Growth of 2D Perovskite Nanowires for Polarized Light Detection. Adv Opt Mater 2019;7:1900039.

[19] Raja SN, Bekenstein Y, Koc MA, et al. Encapsulation of Perovskite Nanocrystals into Macroscale Polymer Matrices: Enhanced Stability and Polarization. ACS Appl Mater Inter 2016;8:35523-35533.

[20] He J, Towers A, Wang YN, et al. In situ synthesis and macroscale alignment of $CsPbBr_3$ perovskite nanorods in a polymer matrix. Nanoscale 2018;10:15436-15441.
